# The *miR156*-Targeted *SQUAMOSA PROMOTER BINDING PROTEIN* (*PmSBP*) Transcription Factor Regulates the Flowering Time by Binding to the Promoter of *SUPPRESSOR OF OVEREXPRESSION OF CO1* (*PmSOC1*) in *Prunus mume*

**DOI:** 10.3390/ijms231911976

**Published:** 2022-10-09

**Authors:** Xue Yong, Tangchun Zheng, Yu Han, Tianci Cong, Ping Li, Weichao Liu, Aiqing Ding, Tangren Cheng, Jia Wang, Qixiang Zhang

**Affiliations:** Beijing Key Laboratory of Ornamental Plants Germplasm Innovation & Molecular Breeding, National Engineering Research Center for Floriculture, Beijing Laboratory of Urban and Rural Ecological Environment, Engineering Research Center of Landscape Environment of the Ministry of Education, Key Laboratory of Genetics and Breeding in Forest Trees and Ornamental Plants of the Ministry of Education, School of Landscape Architecture, Beijing Forestry University, Beijing 100083, China

**Keywords:** *Prunus mume*, *SQUAMOSA PROMOTER BINDING PROTEIN*, *miR156*, *SUPPRESSOR OF OVEREXPRESSION OF CO1*, flowering time

## Abstract

*Prunus mume*, a famous perennial ornamental plant and fruit tree in Asia, blooms in winter or early spring in the Yangtze River area. The flowering time directly determines its ornamental and economic value, so it is of great significance to study the molecular mechanism of flowering time. *SQUAMOSA PROMOTER BINDING PROTEIN* (*SBP*), often regulated by *miR156*, is an important flowering regulator, although its function is unknown in *P. mume*. Here, 11 *miR156* precursors were analyzed and located in five chromosomes of the *P. mume* genome. The expression pattern showed that *PmSBP1*/*6* was negatively correlated with *miR156*. The promoters of *PmSBP1*/*6* were specifically expressed in the apical meristem. Overexpression of *PmSBP1*/*6* in tobacco promoted flowering and changed the length ratio of pistil and stamen. Moreover, *PmSBP1* also affected the number and vitality of pollen and reduced the fertility of transgenic tobacco. Furthermore, ectopic expression of *PmSBP1/6* caused up-regulated expression of endogenous *SUPPRESSOR OF OVEREXPRESSION OF CO1* (*NtSOC1*). The yeast-one hybrid assay showed that PmSBP1 was bonded to the promoters of *PmSOC1s*. In conclusion, a miR156-PmSBP1-PmSOC1s pathway was formed to participate in the regulation of flowering time in *P. mume*, which provided references for the molecular mechanism of flowering time regulation and molecular breeding of *P. mume.*

## 1. Introduction

Flowering time is a very important agronomic trait, which directly determines the ornamental value and economic benefits of ornamental plants. Plant flowering marks the end of vegetative growth and the beginning of reproductive growth, which is a very complex and important link in the process of plant growth and development. It is not only affected by external environmental factors (light, temperature, etc.) but also regulated by internal physiological factors. So far, six pathways regulating the flowering time of plants have been identified. These pathways include environmental factor pathways: photoperiod pathway, vernalization pathway, and temperature pathway; and the physiological factor pathways: autonomous pathway, gibberellin pathway, and aging pathway. Flowering time is jointly regulated by these pathways, which can affect each other and integrated by a series of genes, such as *SUPPRESSOR OF OVEREXPRESSION OF CO1* (*SOC1*), *LEAFY* (*LFY*), *FLOWERING LOCUS T* (*FT*), *CONSTANS* (*CO*), *FLOWERING LOCUS C* (*FLC*), etc. [1].

The aging pathway is based on the phenomenon that plants must grow to a certain age before the commencement of reproductive growth. The aging pathway is mainly regulated by *microRNA156* (*miR156*) and its target gene *SQUAMOSA PROMOTER BINDING PROTEIN-LIKE* (*SPL*), which was also named *SQUAMOSA PROMOTER-BINDING PROTEIN (SBP)*. The expression of *miR156* and *SPL* genes is negatively correlated. For example, from the juvenile to adult phase of *Arabidopsis thaliana*, the expression level of *miR156* was gradually decreased, while the expression level of its target gene *AtSPL*, increased with the increase of age, and reached a high level in adulthood [2,3,4]. In addition, their expression patterns were conservative in many species [5,6,7,8]. The miR156-SPL mode plays an important role in the flowering process of plants. In *Arabidopsis*, constitutive expression of *miR156* could prolong the vegetative growth, and overexpression of its target gene *SPL* resulted in early flowering [3,4,9,10,11]. Furthermore, the aging pathway can mediate photoperiod, gibberellic acid, and endogenous flowering pathways, and co-regulate plant flowering by affecting flowering integrators, such as *SOC1* [12,13], *CO* [14], *FT* [2,15], *AP1*, *FUL*, and *LFY* [11,16]. However, *AtSPL8*, a member of SBP-box genes in *Arabidopsis*, does not affect the flowering time, but affects the development of the flower organs and reduces the fertility of plants [17].

Mei (*Prunus mume*), which belongs to *Prunus* in Rosaceae, is an important perennial woody ornamental plant and fruit tree. *P. mume* is widely spread in temperate regions of Asia and has been cultivated for more than 3000 years in China. The early flowering determines the ornamental, economic, and cultural value of Mei. So far, some flowering-related genes in *P. mume* have been studied, such as *PmSOC1* [18], *PmSVP* [19], and *PmLFY* [20]. The SBP-box genes have been identified in *P. mume* [21]. However, the molecular mechanism of *PmSBPs* in flowering regulation is still not clear. Here, two members of the SBP-box gene family (*PmSBP1* and *PmSBP6*) were cloned, and their expression patterns, gene function, and regulation mechanism were studied. The outcomes, thus, lay a foundation for clarifying the molecular mechanism of flowering time regulation of *P. mume* and other *Prunus* species.

## 2. Results

### 2.1. Chromosomal Location, Phylogenetic Analysis and Sequence Analysis of miR156 from P. mume

In *P. mume*, the precursors of *pmu-miR156* were encoded by 11 genomic sequences (*pmu-MIR156a-k*), which were located in five chromosomes (Supplementary Figure S1A) and cut into four types of mature sequences (*pmu-miR156a–d*, *e–f*, *g–j*, *k*). An ML phylogenetic tree of 36 precursor sequences (11 from *P. mume*, 10 from *Arabidopsis*, and 15 from tobacco) was built and the miR156 family was divided into three subgroups (Supplementary Figure S1B). Among them, the *pmu-MIR156i*/*j*/*g*/*h* were clustered with *ath-MIR156a–f* and *nta-MIR156c–i*, and the *pmu-MIR156a*/*b*/*c*/*d*/*f*/*k* were clustered with *ath-MIR157a–d* and *nta-MIR156a*/*b*/*j–o*, but the *pmu-MIR156e* was a subgroup alone. Sequence alignment showed that only two differentiated bases were found between the *miR156* mature sequences from *P. mume*, *Arabidopsis*, and tobacco (Supplementary Figure S1C). The *pmu-miR156f* can fully bind to the *miR156* site on the mRNA of *PmSBP1*, and they are located on the same chromosome.

### 2.2. Expression Patterns of PmSBP1/6 and miR156f during the Flower Development Period

**Figure 1:**
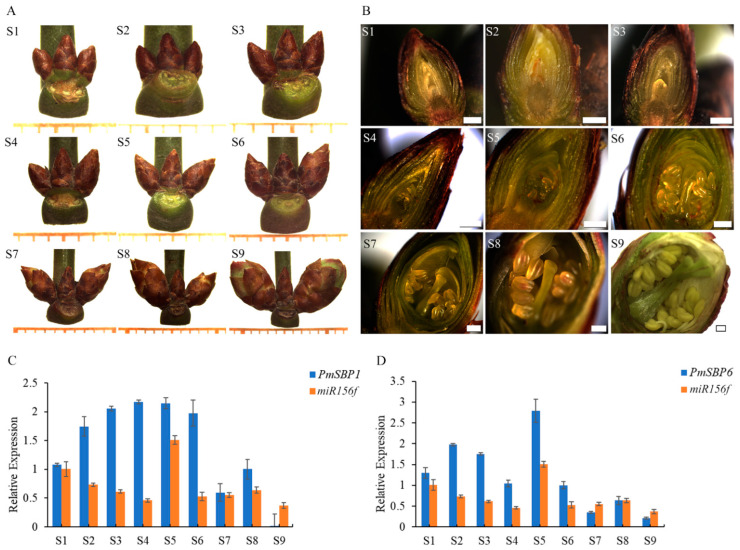
The flower development stages and the expression patterns of *PmSBP1*/*6* and *miR156f*. (**A**,**B**) Flower buds at different development stages. S1: flower primordium forming stage, S2: sepal forming stage, S3: petal forming stage, S4: stamen forming stage, S5: pistil forming stage, S6: anther and ovule forming stage, S7: morphological differentiation finished stage, S8: alabastrum intumescence stage, S9: flower upcoming to bloom. S1–S7 belong to the flower bud morphological differentiation phase, and S6–S9 belong to the flower bud dormancy phase, the two phases have overlapping parts. The minimum scale in (**A**) = 1 mm, and the scale bar in (**B**) = 400 μm. (**C**,**D**) Expression patterns of *PmSBP1, PmSBP6* and *miR156f* during the flower development period.

The flower development period of *P. mume* consists of two phases with overlapping parts: flower bud morphological differentiation and dormancy, which were divided into nine stages (S1–S9): flower primordium forming stage (S1), sepal forming stage (S2), petal forming stage (S3), stamen forming stage (S4), pistil forming stage (S5), anther and ovule forming stage (S6), morphological differentiation finished stage (S7), alabastrum intumescence stage (S8), and flower upcoming to bloom stage (S9) (Figure 1A,B). Among them, S1–S7 belong to the flower bud morphological differentiation phase, and S6–S9 belong to the flower bud dormancy phase. To verify the function of *PmSBP1*/*6* and the regulation relationship with *miR156f*, their expression patterns in flower development were analyzed. The expression level of *PmSBP1* gradually increased (S1–S4) and remained stable (S4–S6), but decreased sharply when flower bud morphological differentiation was completed (S7), then recovered slightly at S8 and then maintained a very low level before blooming (S9) (Figure 1C). The expression level of *PmSBP6* increased first (S1–S2) and decreased gradually from S2 to S4, then suddenly raised at pistil forming stage (S5) and then dropped when flower bud morphological differentiation was completed (S7), and maintained at a low level (S8–S9) (Figure 1D). In general, *PmSBP1*/*6* shows relatively high expression in the flower bud morphological differentiation phase, but low expression after differentiation. On the other hand, the expression level of *pmu-miR156f* decreased from S1 to S4, but suddenly increased at S5, then fell back and remained at a comparatively stable level (S6–S9). In conclusion, the expression patterns of *PmSBP1*/*6* and *pmu-miR156f* were negatively correlated.

### 2.3. The Activity Analysis of PmSBP1/6 Promoters

**Figure 2:**
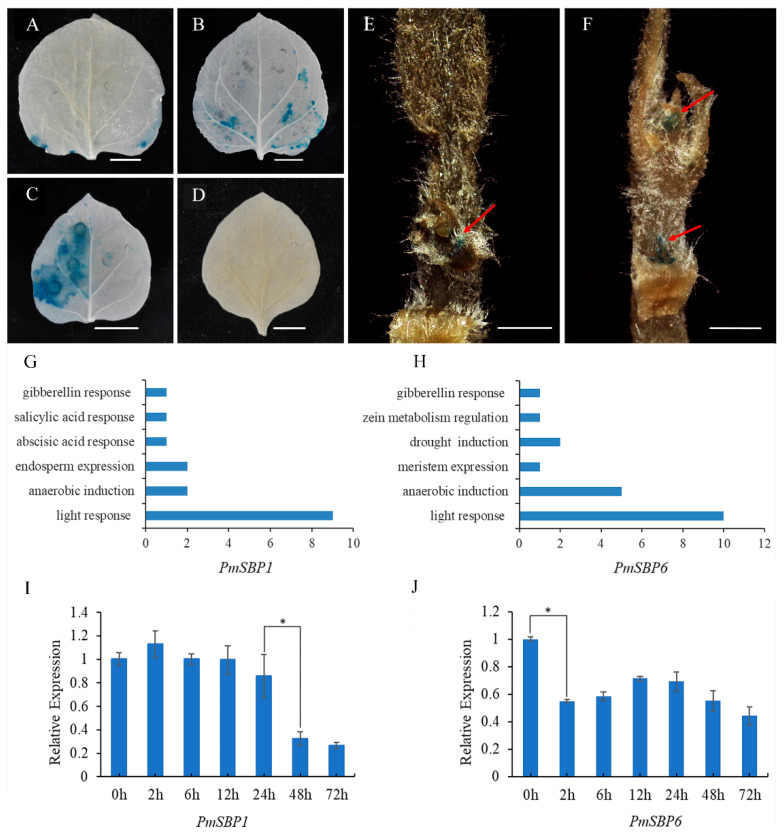
Analysis of the *PmSBP1*/*6* promoter function and their response to GA3. (**A**,**B**) The activity of the *PmSBP1*/*6* promoters. (**C**) The *35S::GUS* positive control. (**D**)The negative control without any vector. The scale bar in (**A**–**D**) is 1 cm. (**E**,**F**) The spatial expression profile of *PmSBP1*/*6*. The red arrows indicate the location of GUS staining. The scale bar is 1 mm. (**G**,**H**). The promoter *cis*-elements composition of *PmSBP1*/*6*. (**I**,**J**) The expression of *PmSBP1*/*6* under GA3 treatment. The asterisk indicates significant differences (*p* < 0.01).

To verify the activity of the *PmSBP1*/*6* promoters, the 2000 bp upstream sequences of *PmSBP1*/*6* were cloned and fused with the *β-glucuronidase* (*GUS*) gene and transiently transformed into the *N. benthamiana* leaves by *A. tumefaciens*. Finally, clear blue spots were observed in the tobacco leaves with *PmSBP1*/*6* promoters (Figure 2A,B) compared with the control (Figure 2C,D). For further study of the tissue spatial expression profile of *PmSBP1*/*6* promoters, the promoters with *GUS* were stably overexpressed in tobacco. In the *PmSBP1*-promoter transgenic tobacco, obvious blue tissues were detected in the apical meristem (Figure 2E). While in the *PmSBP6*-promoter transgenic tobacco, the blue tissues were detected in both the apical meristem and lateral bud (Figure 2F).

### 2.4. The Promoter Cis-Elements Composition of PmSBP1/6 and Their Response to GA3

The promoter *cis*-elements can affect the gene expression. The cloned promoter sequences of *PmSBP1*/*6* (about 2000 bp) were predicted on the PlantCARE website (Supplementary Table S2 and Supplementary Data S1). Statistical results of *cis*-elements showed that gibberellin response elements, anaerobic induction elements, and light response elements were contained in the promoters of both *PmSBP1* and *PmSBP6* (Figure 2G,H). In addition, the promoter of *PmSBP6* also contained meristem expression elements (Figure 2H). To analyze whether the expression of *PmSBP1*/*6* is regulated by gibberellin, the qRT-PCR was carried out with the flower buds treated with 100 mg/L GA3 solutions. The expression patterns of *PmSBP1* and *PmSBP6* are shown in Figure 2I,J. The expression level of *PmSBP1* and *PmSBP6* significantly decreased after gibberellin treatment for 48 h and 2 h, respectively (Figure 2I,J).

### 2.5. Subcellular Localization of PmSBP1/6 Proteins

**Figure 3:**
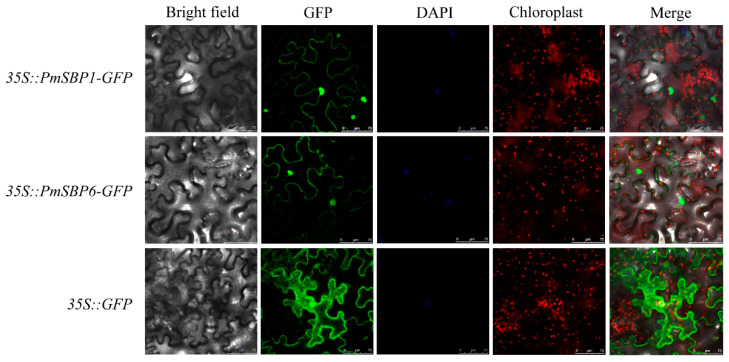
Subcellular localization of PmSBP1/6. The green, blue, and red fluorescence represent GFP fusion protein, nucleus, and chloroplast positions. The merge pictures were made of bright field, GFP, DAPI, and chloroplast pictures. The scale bar = 75 μm.

The subcellular localization of PmSBP1/6 was detected by the transient transformation of the PmSBP1/6-GFP fusion proteins into tobacco leaves. In the control *35S::GFP*, the GFP fluorescence signals were examined in the cytoplasm and nucleus. In the *35S::PmSBP1/6-GFP*, although the GFP fluorescence signals were detected mainly in the nucleus and cytoplasm, the fluorescence intensity is weaker than the control (Figure 3).

### 2.6. Overexpression of PmSBP1/1tb and PmSBP6 in Tobacco Promotes Flowering and Affects Fertility

**Figure 4:**
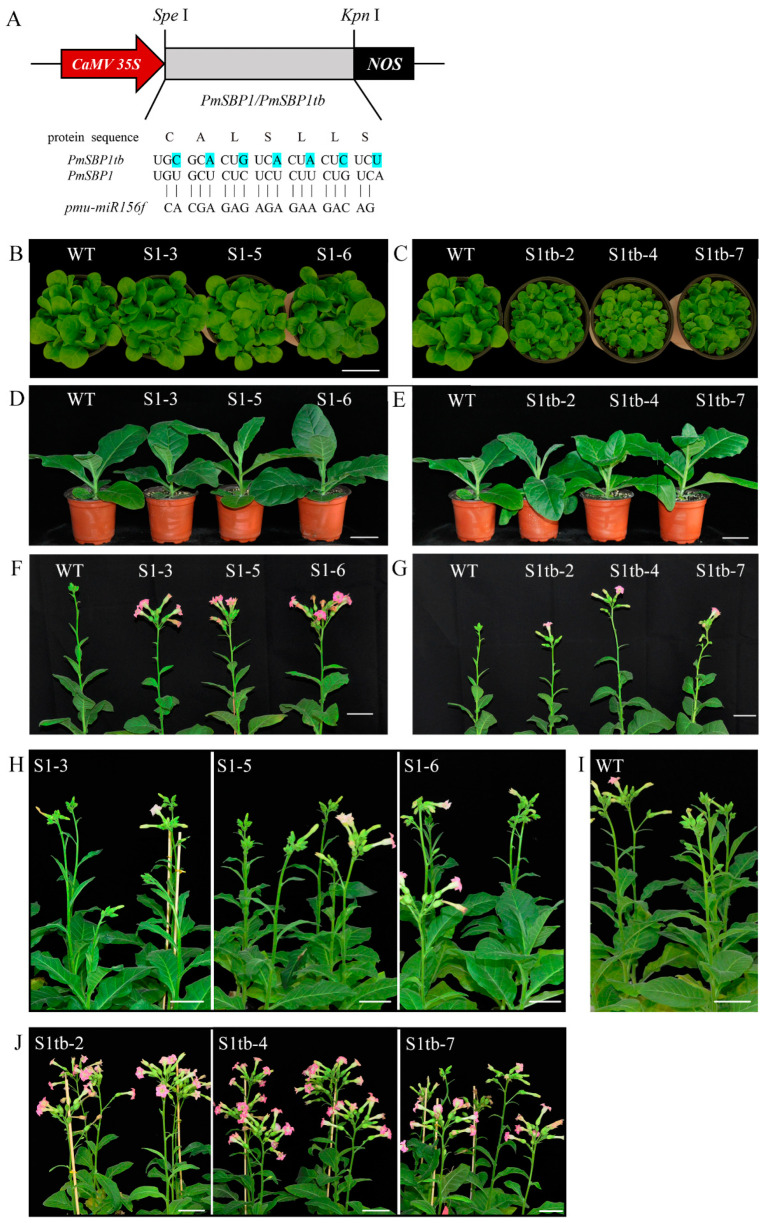
Overexpression of *PmSBP1/1tb* in tobacco. (**A**) The structure of *PmSBP1/1tb* recombinant vectors. (**B**–**E**) The phenotype of *PmSBP1* and *PmSBP1tb* transgenic plants in the vegetative growth period (50 d and 80 d). (**F**,**G**) The flowering time of *PmSBP1* and *PmSBP1tb* transgenic plants under short-day conditions (100 d). (**H**–**J**) The flowering time of *PmSBP1, PmSBP1tb* transgenic plants and WT under long-day conditions (110 d). The scale bar = 5 cm.

**Figure 5:**
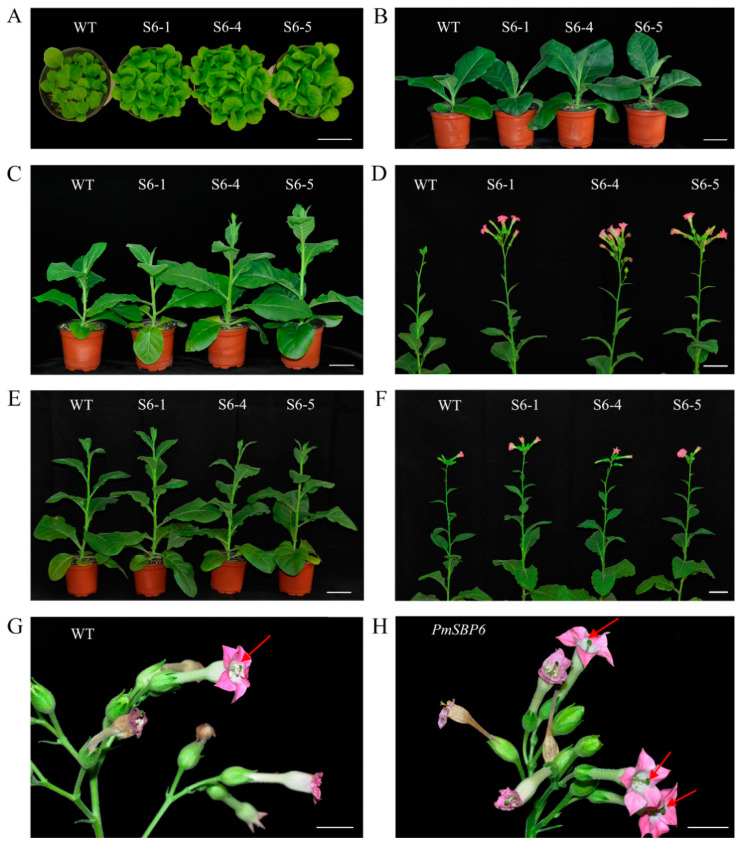
The phenotype of *PmSBP6* transgenic plants. (**A**,**B**) The vegetative growth period of *PmSBP6* transgenic plants (50 d and 80 d). (**C**,**D**) The flowering time of *PmSBP6* transgenic plants grown in short-day conditions (90 d and 100 d). (**E**,**F**) The flowering time of *PmSBP6* transgenic plants grown in long-day conditions (100 d and 110 d). (**G**,**H**) The flower phenotype of WT and the *PmSBP6* transgenic tobacco. The red arrows indicate the relative height of stigma and stamen. The scale bar = 1 cm.

The *PmSBP1*, *PmSBP1tb*, and *PmSBP6* were overexpressed in tobacco under *CaMV35S*. To confirm the function of *PmSBP1* and *miR156*, the *miR156* site of *PmSBP1* was synonymously mutated and named *PmSBP1tb*. The schematic map of *PmSBP1*/*1tb* recombinant vectors is shown in Figure 4A. More than 10 transgenic lines of each gene were obtained and confirmed by a reverse transcription polymerase chain reaction (RT-PCR) assay, and three lines were selected for subsequent analyses. In the juvenile period of transgenic T_3_ generation plants, the vegetative growth of the *35S::PmSBP1tb* transgenic seedlings was significantly less than that of the wild type (WT) (Figure 4C), but the *35S::PmSBP1* transgenic lines were slightly larger than that of the WT (Figure 4B). Besides, the vegetative growth of the three *35S::PmSBP6* transgenic lines was also a little larger than that of the WT (Figure 5A). As plants grew older, the difference in vegetative growth between all the transgenic seedlings and wild type gradually disappeared (Figure 4D,E and Figure 5B).

The flowering time of transgenic plants was different when they under different photoperiod conditions. In short-day conditions, both *PmSBP1* (Figure 4F), *PmSBP1tb* (Figure 4G), and *PmSBP6* (Figure 5C,D) transgenic plants bloomed earlier than WT. In addition, the flowering time of *PmSBP1* transgenic plants was earlier than that of *PmSBP1tb*. While under the long-day conditions, the flowering time of *PmSBP1* (Figure 4H) and *PmSBP6* (Figure 5E, F) transgenic lines was similar to that of WT (Figure 4I). Interestingly, the flowering time of the *PmSBP1tb* transgenic lines was significantly earlier than that of WT (Figure 4J).

**Figure 6:**
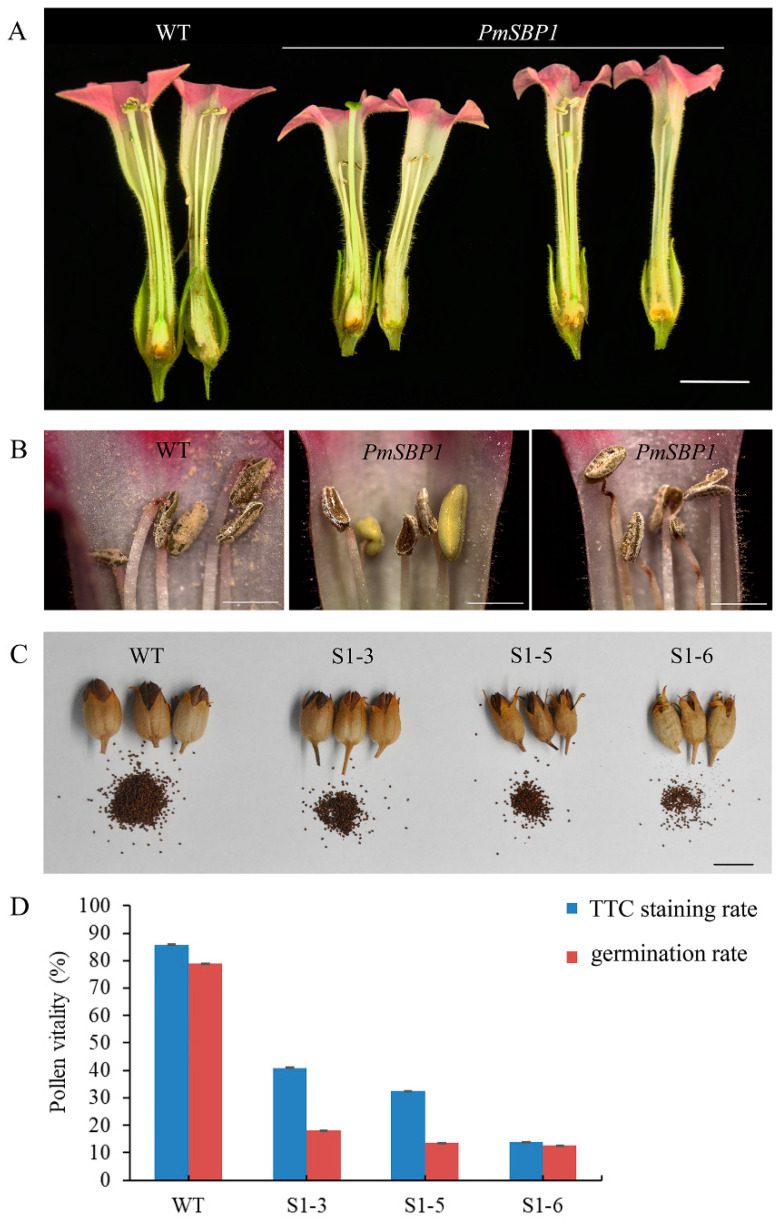
The phenotype of reproductive organs and pollen vitality of *PmSBP1* transgenic tobacco. (**A**) The floral organs of *PmSBP1* transgenic plants. (**B**) The anther morphology of *PmSBP1* transgenic plants. (**C**) The pod size and seed number from one pod in the *PmSBP1* transgenic plants. (**D**) The pollen vitality of *PmSBP1* transgenic plants. The scale bar in (**A**,**C**) = 1 cm. The scale bar in (**B**) = 2 mm.

In the reproductive growth stage, the *35S::PmSBP1tb* transgenic tobacco was normal, but the *35S::PmSBP1* and *35S::PmSBP6* transgenic tobacco were different from the wild-type tobacco. In the wild-type tobacco, the length of style and filaments were the same, allowing them to be self-bred and bear fruit (Figure 5G). While in the *35S::PmSBP1* (Figure 6A) and *35S::PmSBP6* (Figure 5H) transgenic tobacco, the length of style and filaments were inconsistent. In the *35S::PmSBP1* transgenic tobacco, the length ratio of style to filament showed two phenotypes: one was the high-style (the style was higher than the filament) and the other was the short-style (the style was shorter than the filament). Among them, the high-style flower was the main phenotype (96%), with smaller size, withered anthers, less pollen, and fast-drying filaments (Figure 6A,B), which could not be self-pollinated. The pods became smaller and the seed number in one pod was less after artificial self-pollination (Figure 6C). The pollen viability of the high-style flower was tested by TTC staining and pollen germination in vitro. As shown in Figure 6D and Supplementary Figure S2, in the wild-type tobacco and three *35S::PmSBP1* transgenic lines S1-3, S1-5, and S1-6, the pollen staining rates were 85.8%, 41%, 32.5%, and 13.8%, respectively, and the pollen germination rates were 78.8%, 18.2%, 13.7%, and 12.5%, respectively. In short, the results of the two methods showed that the pollen viability of *35S::PmSBP1* transgenic plants was lower than that of WT. The flower phenotype in the *35S::PmSBP6* transgenic tobacco was partly different from the *35S::PmSBP1* transgenic tobacco. In the *35S::PmSBP6* transgenic tobacco, all the flowers showed a high-style phenotype with normal size, but the flowers could bear fruit normally after artificial self-pollination (Figure 5H).

### 2.7. Expression Pattern of Endogenous Flowering-Related Genes in PmSBP1/1tb and PmSBP6 Transgenic Tobacco

**Figure 7:**
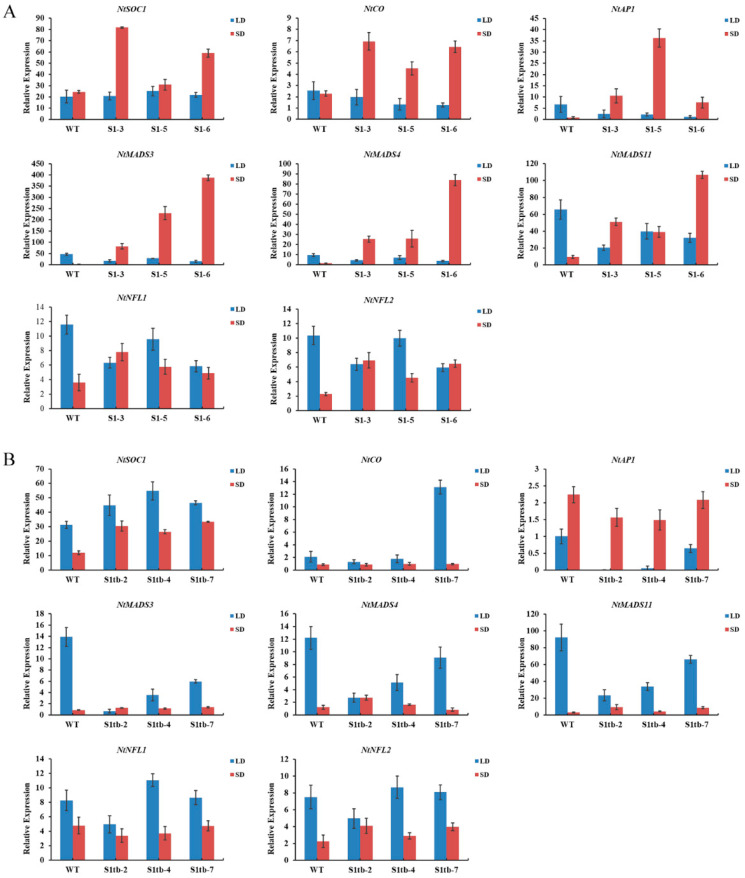
The relative expression of endogenous flowering-related genes in *PmSBP1* and *PmSBP1tb* transgenic tobacco under long and short conditions. (**A**) The expression of flowering-related genes in *PmSBP1* transgenic tobacco. S1-3, S1-6, and S1-7 were three transgenic lines. (**B**) The expression of flowering-related genes in *PmSBP1tb* transgenic tobacco. S1tb-2, S1tb-4, and S1tb-7 were three transgenic lines.

**Figure 8:**
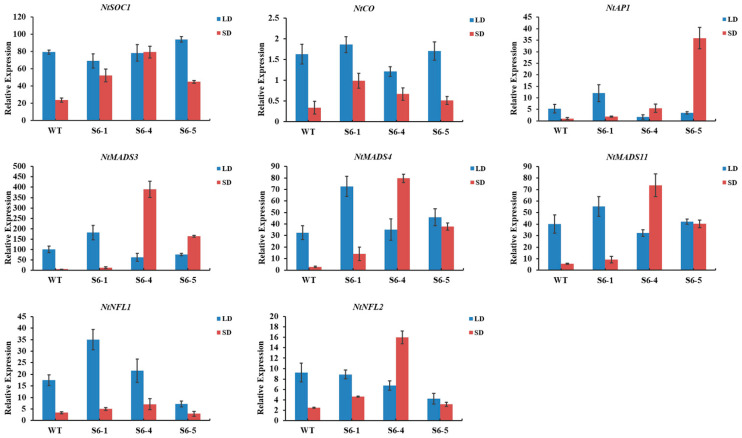
The relative expression of endogenous flowering-related genes in *PmSBP6* transgenic tobacco under long and short conditions. S6-1, S6-4, and S6-5 were three transgenic lines.

To investigate the early flowering transgenic tobacco, the expression level of eight endogenous flowering-related genes (*NtSOC1*, *NtCO*, *NtAP1*, *NtMADS3*, *NtMADS4*, *NtMADS11*, *NtNFL1*, and *NtNFL2*) in the shoot tip of tobacco (90 d in short-day conditions, 100 d in long-day conditions) was detected by qRT-PCR. The expression level of these eight endogenous flowering-related genes in the three *PmSBP1* transgenic tobacco lines is shown in Figure 7A. In short-day conditions, their expression was higher than that of the wild-type tobacco; while in long-day conditions, the expression level was lower than that of wild-type tobacco, except for *NtSOC1* showing similar expression to wild-type tobacco. As shown in Figure 7B, the expression level of the endogenous *NtSOC1* in the *PmSBP1tb* transgenic tobacco was increased under both short-day and long-day conditions. In short-day conditions, the expression of the other seven flowering-related genes was similar to WT. In long-day conditions, the expression of *NtCO* only increased sharply in the *PmSBP1tb* transgenic tobacco line S1tb-7; however, it changed slightly in the other two lines. The expression level of *NtAP1*, *NtMADS3*, *NtMADS4*, and *NtMADS11* in all three lines decreased; however, the expression of *NtNFL1* and *NtNFL2* fluctuated marginally. As shown in Figure 8, the expression level of these eight flowering-related genes in the three *PmSBP6* transgenic lines under short-day conditions was higher than that of WT. Under long-day conditions, their expression levels in the three lines fluctuated up and down at the relative expression value of WT. The expression level of *NtSOC1* was directly proportional to the phenotype of early flowering in these transgenic plants.

### 2.8. PmSBP1 Binding to the PmSOC1s Promoters

**Figure 9:**
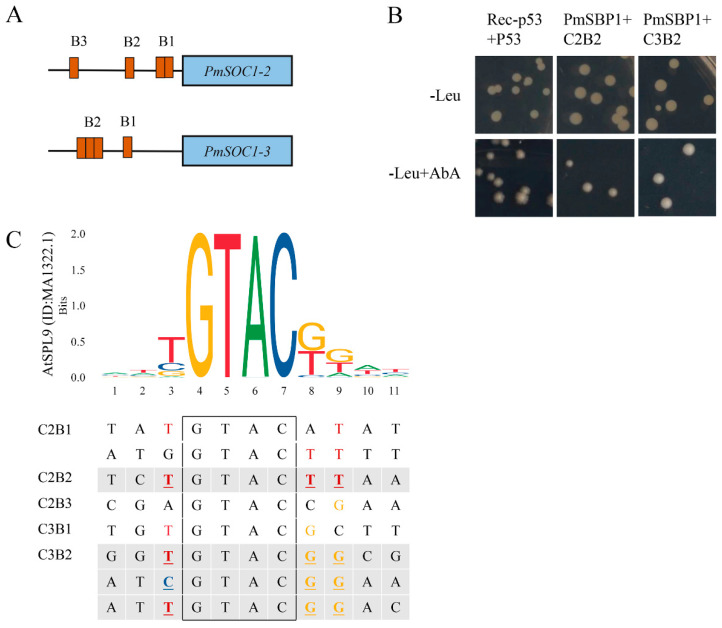
PmSBP1 binding to the *PmSOC1s* promoter. (**A**) The SBP binding sites on the *PmSOC1s* promoters. (**B**) The Y1H results of PmSBP1 and the *PmSOC1s* promoter fragments. (**C**) The analysis of the SBP-binding fragments in *PmSOC1s* promoters. The black box is the core component ‘GTAC’ of the SBP-binding fragments. The sequence logo of the SBP-domain in AtSPL9 (ID: MA1322.1) was downloaded from JASPAR (http://jaspar.genereg.net, accessed on 16 September 2022).

To verify the regulatory relationship between *PmSBP1* and *PmSOC1s*, the promoter sequences of *PmSOC1s* (about 850 bp) were cloned and analyzed (Supplementary Data S1). Among them, except for *PmSOC1-1*, the promoters of *PmSOC1-2* and *PmSOC1-3* contained four SBP binding sites ‘GTAC’ respectively (Figure 9A). The 100 bp fragments of *PmSOC1s* promoters containing SBP binding sites were used as baits. The B1 fragment of *PmSOC1-2* (C2B1) and the B2 fragment of *PmSOC1-3* (C3B2) contained two and three SBP binding sites, respectively. The interactions between PmSBP1 and the fragments of *PmSOC1s* promoters were detected by yeast one-hybrid (Figure 9B). All transformed yeast grew normally on the SD/-Leu solid medium. As shown in Figure 9B, PmSBP1 could bind to the B2 fragments of *PmSOC1-2* (C2B2) and *PmSOC1-3* promoters (C3B2) but could not associate with the other fragments (C2B1, C2B3, and C3B1). This result suggested that PmSBP1 could activate *PmSOC1-2* and *PmSOC1-3* by binding to the B2 sites of their promoters. Furthermore, we compared the eight SBP-binding fragments containing the core ‘GTAC’ (11 bp) with the SBP-binding sequence of *Arabidopsis* AtSPL9 (ID: MA1322.1, homologous with PmSBP1), and found that the three bases adjacent to the core ‘GTAC’ in C2B2 and C3B2 were consistent with the high-frequency bases in the SBP-binding sequence of AtSPL9 (Figure 9C), which indicated that the three bases adjacent to ‘GTAC’ were critical for the binding with PmSBP1.

## 3. Discussion

Plant microRNAs (miRNAs), 19–25 nt long, are highly conserved small non-coding RNAs, and play important role in juvenile-to-adult phase transition and flowering time in *Arabidopsis* [22,23]. The *miR156* is quite conservative among plant species [24]. In this study, the sequence alignment results showed that the *miR156* mature sequences in *P. mume*, *Arabidopsis* and tobacco were indeed highly conserved, with only two SNPs (Supplementary Figure S1A). The miR156-SPL is the famous age pathway; *SPL* is the target gene of *miR156*, and is regulated by *miR156* mainly through complementation base-pairing at the post-transcriptional level [23]. In our previous study, the *miR156*-mediated *PmSBPs* mRNA cleavage was detected by 5′-RACE [25].

In *Arabidopsis*, there are 16 *AtSPLs*, which are clustered into two clades: clade I contains two subclades *AtSPL7* and *AtSPL1/12/14/16*; clade II contains four subclades *AtSPL3/4/5, AtSPL2/10/11, AtSPL9/15/6/13*, and *AtSPL8*, and all members in clade II have *miR156/157* sites except for *AtSPL8* [26]. In this study, *PmSBP1* were clustered with the *AtSPL9*/*15*, and *PmSBP6* were clustered with *AtSPL3*/*4*/*5* together with *PmSBP7*/*8* [21]. Both *PmSBP1* and *PmSBP*6 have *miR156* binding sites, but the difference is that the *miR156* binding site of *PmSBP1* is in the coding region, while the *miR156* binding site of *PmSBP6* is in the 3′ UTR region [25]. Here, the expression patterns of *pmu-miR156f* and *PmSBP1/6* during the flower development period were negatively correlated.

The spatiotemporal expression pattern of genes is closely related to gene function. So far, the expression pattern of *SBP* in the flower development period has been studied in some perennial plants. The expression profile of *AtSPL9/15* homologous genes in some species is as follows. In loquat, the expression of *EjSPL9* decreased with the development of flower bud [27]. In chestnut, the expression level of *CmSPL9* was increased with the development of flower bud [28], and so does the *JrSBP23* in walnut [29]. In *Betula luminifera*, *BlSPL8* was highly expressed in the early and middle stages of male inflorescence [30]. Unlike them, the expression of *PmSBP1* was relatively stable and high in the flower bud morphological differentiation phase of *P. mume*, which indicated that *PmSBP1* may play a vital role in flower bud differentiation. The expression pattern of *AtSPL3/4/5* homologous genes in other species is as follows. In loquat, the expression of *EjSPL3* and *EjSPL 4* reached the peak at flower bud initiation, the expression level of *EjSPL4* and *EjSPL5* was suddenly raised in the middle stage of flower bud development [27]. The *BlSPL15* of *Betula luminifera* was significant highly expressed in the early stage of female inflorescence [30]. But partly like *EjSPL4*, *EjSPL5* and *BlSPL15*, in this study, the expression level of *PmSBP6* was significantly high at the stamen differentiation stage (S5), which implied that it may participate in the stamen differentiation or regulate fertility. Different expression patterns in different species indicated that they may have different functions during flower bud development.

The promoter is the switch of the gene, which can regulate the gene expression, or bind to the transcription factors to start or close the gene expression. In this study, the 2000 bp promoter sequences of *PmSBP1*/*6* were cloned, both had typical promoter core structure regions, and the GUS staining results showed they had driven activity. Some fragment deletions were found in the cloned promoter sequences of *PmSBP1*/*6* when compared with the genome sequence of wild *P. mume*. This may be the evolutionary result of the changes in environmental factors during the long-term cultivation process. In addition, the two promoter sequences contained multiple *cis*-acting elements, such as the light response elements, the anaerobic inducing elements, hormone response elements, and the endosperm expression element or meristem expression element. This suggested that *PmSBP1* and *PmSBP6* can be regulated by external environmental stimuli (light, water, and hormones), and may be expressed in some specific plant tissues. Besides, the exogenous GA3 downregulated the expression of *PmSBP1*/*6*, which was similar to the result of *CmSPL9* in chestnut [28]. In addition, the tissue spatial expression profile showed that the *PmSBP1* promoter was initiated in the apical meristem, and the *PmSBP6* promoter was initiated in both the apical meristem and lateral bud. This displayed that they may play an important role in the initiation of flower buds.

Recently, the function of *AtSPLs* in clade II of *Arabidopsis* was extensively studied. *AtSPL8* functions in both the male and female fertility of *Arabidopsis* by affecting the anther development [17,31,32,33] and gynoecium patterning [33]. Based on the function research results, the other ten *AtSPLs* with *miR156*/*157* sites are divided into three groups. Group 1 contains *AtSPL2*/*9*/*10*/*11*/*13*/*15*, which play role in both the transition from childhood to adulthood and from nutrition to reproduction, and *AtSPL9*/*13*/*15* [34] play more important roles than *AtSPL2*/*10*/*11* [10]. Group 2 contains *AtSPL3*/*4*/*5*, which can promote the transformation of flower meristem, but play a minor role in the change of vegetative stage or flower induction [16,35]. Group 3 contains *AtSPL6*, which does not play a major role in shoot morphogenesis, but may play an important role in some physiological processes [11]. As is known, the miR156-SPL is an important regulatory model in plant growth. Overexpression of *miR156* in *Arabidopsis* prolongs childhood, while silencing *miR156* makes plants mature early [3]. In *Arabidopsis* [2], tobacco [36], and several other plants [6,30,37], the content of *miR156* was high and the content of *SPL* was low in childhood. With the increase of plant age, the content of *miR156* decreased and the content of *SPL* increased. In addition, their expression can be regulated by each other [3,9].

In our study, *PmSBP1* and its synonymous mutation *PmSBP1tb* and *PmSBP6* were constitutively overexpressed in tobacco and showed their role in the regulation of plant growth, flowering time, and reproductive organ development. As our results showed, in the childhood stage, the *35S::PmSBP1tb* transgenic tobacco was significantly smaller than that of the wild-type tobacco (Figure 4C), while the *35S::PmSBP1* transgenic seedlings were only slightly larger than the wild-type tobacco (Figure 4B). This may be related to the regulatory balance between the content of excessive SBP transcript and *miR156*. Unexpectedly, the *35S::PmSBP6* transgenic seedlings were also slightly larger than WT in the childhood stage (Figure 5A). Like *PmSBP1tb*, The CDS region of *PmSBP6* does not contain a *miR156* binding site, which cannot be regulated by *nta-miR156* in tobacco, but they do have opposite phenotypes (Figure 4B and Figure 5A). We speculated that there was still a regulatory relationship between *PmSBP6* and *miR156* after removing the 3′ UTR sequence (containing *miR156* binding site); however, further research should be conducted to confirm the new findings.

The development of floral organs is very important for the sexual reproduction of plants, especially the stamen and pistil. In *Arabidopsis*, the *AtSPL8* (without *miR156* binding site) and other *AtSPLs* (with *miR156* binding site) are necessary for the production of fully fertile flowers [23,26]. The knocking out of *AtSPL8* resulted in abnormal anther development [17], but the overexpressing of *AtSPL8* caused anther non-dehiscence [32]. When overexpressed *miR156* in the *spl8* mutant, the plant showed complete male sterility, but the overexpression of other *AtSPL* with *miR156* binding sites in the *spl8* mutant can alleviate the semi-sterile phenotype to a certain extent [26]. Another *miR156* targeted gene *AtSPL2* can affect plant fertility by affecting pollen production and fertilization rate. The fertility of both the gain-of-function mutant *35S::SPL2SRDX* and the loss-of-function mutant *spl2* decreased, and the fertility of the loss-of-function mutant was lower [38]. In this research, the overexpression of *PmSBP1* (homologous to *AtSPL9/15*) and *PmSBP*6 (homologous to *AtSPL3/4/5*) in tobacco caused changes in the length of style and filament, and led to changes in the pollination mode, especially *PmSBP1*, which caused pollen abortion by reducing the number and vitality of pollen. However, overexpression of the *PmSBP1tb* gene neither caused changes in flower organs nor affected the pollen number and activity, and the fertility of *PmSBP1tb* transgenic plants was not affected. This indicated that both *PmSBP1* and *PmSBP6* can change the length ratio of style to the filament and affect the pollination mode, and the *miR156* locus of *PmSBP1* is indispensable in affecting plant fertility. Similar to *Arabidopsis AtSPL8* [23,26] and *AtSPL2* [38], all these genes affect plant fertility, but in different ways. Among these *SBPs* from different species, those not clustered together by phylogenetic analysis also have similar functions, this may be due to the species evolution.

Ectopic expression of *PmSBP1* and *PmSBP6* in tobacco caused early flowering, which was similar to their homologous genes in *Arabidopsis* [15,34,35]. However, in our study, the flowering time of each transgenic plant was not consistent under different photoperiod conditions. The flowering time of *PmSBP1* and *PmSBP6* transgenic tobacco was earlier than that of WT only in short-day conditions, but the flowering time of *PmSBP1tb* transgenic tobacco was earlier in both long-day and short-day conditions. This indicated that *PmSBP1* and *PmSBP6* were affected by *miR156* in promoting flowering in tobacco through mediating the photoperiod pathway. Furthermore, we examined the expression pattern of eight endogenous flowering-related genes in transgenic plants under different photoperiod conditions. Combined with the phenotype and expression level, we found that the expression level of endogenous *NtSOC1* in early flowering transgenic plants was significantly higher than that of wild-typeunder corresponding photoperiod conditions. This suggested that *PmSBP1* and *PmSBP6* can regulate flowering time by regulating *NtSOC1*. Furthermore, the results of the yeast-one hybrid showed that PmSBP1 can regulate *PmSOC1-2* and *PmSOC1-3* by directly binding to their promoters. However, the relationship between PmSBP6 and the promoter of *PmSOC1s* cannot be verified by yeast-one hybrid because the introduction of PmSBP6 makes yeast grow abnormally. In previous reports, *AtSPL3*/*4*/*5* mediated flowering time regulation by cooperating with FT-FD complexes in the photoperiod pathway [15]. Moreover, the *AtSPL3*/*4*/*5* can be regulated by AtSOC1 by directly binding to their promoters [13]. However, the study on how SBP protein regulates flowering time by directly binding to the promoter of *SOC1* has not been reported. Our findings opened a new way for SBP to regulate flowering time and laid a foundation for molecular breeding of *P. mume* and its research in other species.

## 4. Materials and Methods

### 4.1. Plant Materials

*Prunus mume* ‘Sanlun Yudie’, grown in the campus of Beijing Forestry University was used in this study. The flower buds and young leaves were collected for gene and promoter cloning, respectively. The developmental periods of flower buds were identified by hand sectioning and flower buds were collected from July (initiation of flower bud differentiation) to March (flowering) in the next year for expression pattern analysis. To detect the response of *PmSBP1* on gibberellin, the 100 mg/L GA3 was sprayed on the flower buds of cut-off one-year-old branches after pistil formation and during bud dormancy, the buds were sampled after 0 h, 2 h, 6 h, 12 h, 24 h, 48 h, and 72 h. The *Nicotiana tabacum* and *Nicotiana benthamiana* were grown at 25 °C, 16 h light/8 h dark (long-day conditions) or 8 h light/16 h dark (short-day conditions) in the greenhouse. The stem tips of transgenic and wild-type tobacco were sampled for endogenous gene detection at 100 d in short-day conditions and 110 d in long-day conditions. All samples were collected in liquid nitrogen and stored at −80 °C for RNA isolation.

### 4.2. Bioinformatics Analysis of pmu-miR156

The mature sequences and precursor sequences of *pmu-miR156* in *P. mume*, *ath-miR156* in *Arabidopsis*, and *nta-miR156* in *Nicotiana tabacum* were obtained from our previous study [25], the miRbase (www.mirbase.org/ftp.shtmlmL, accessed on 2 June 2021), and published article [39], respectively. The precursor sequences of *pmu-miR156* were aligned to the genome data of *P. mume* by blastn, and the chromosome position was drawn through the MG2Cv2 (http://mg2c.iask.in/mg2c_v2.0, accessed on 2 June 2021). The DNAMAN software was used to analyze the differences of *miR156* mature sequence in *P. mume, Arabidopsis* and tobacco. The phylogenetic tree of the precursor sequences of three species was obtained through the maximum likelihood method in MEGA software after Clustalx alignment.

### 4.3. Cloning and Analysis of PmSBP Gene and Promoter

The total RNA of flower buds was isolated by EASYspin Plus Plant RNA Kit (Aidlab, Beijing, China), and the first strand of cDNA was synthesized by TransScript RT Kit (TIANGEN, Beijing, China). The genome DNA of young leaves was extracted by DNAsecure Plant Kit (TIANGEN). The cDNA was used as a template to amplify the coding sequences (CDS) of *PmSBP1* and *PmSBP6*. The promoter sequences of *PmSBP1/6* (about 2000 bp) and *PmSOC1s* (about 850 bp) were cloned from the genome DNA by specific primers. The primers were designed by Oligo7 and are listed in Supplementary Table S1. The total volume of the 50 μL polymerase chain reaction (PCR) system includes 25 μL PrimeSTAR HS (Premix) (TaKaRa, Beijing, China), 1 μL forward primer, and 1 μL reverse primer, 2 μL cDNA or 1 μL genome DNA. The PCR procedure was 94 °C pre-denaturation for 2 min; 35 cycles of 98 °C for 10 s, 55 °C for 5 s, 72 °C for 90 s; and 72 °C extensions for 10 min. The PCR products were separated from 0.8% agarose gel using TIANgel Midi Purification Kit (TIANGEN) and cloned into the pCloneEZ-NRS-Omni-Amp/HC vector (Clone Smarter, Houston, TX, USA) and transformed into *Escherichia coli* DH5α for sequencing (TIANGEN). The *cis*-acting element composition of the *PmSBP1*/*6* (about 2000 bp) promoter sequences was predicted on the PlantCARE website [40].

### 4.4. Expression Pattern Analysis

The total RNA of flower buds in different flower development stages and treated with GA3, and the stem tip of tobacco was isolated by the EASYspin Plus Plant RNA Kit (Aidlab). The FastQuant RT Kit (with gDNase) (TIANGEN) was used to synthesize the first strand of cDNA, and the SYBR Premix ExTaq II (TaKaRa) was used for qRT-PCR following the instructions. The miRNA RT/qPCR Detection kit (Aidlab) was used to synthesize cDNA and complete Poly(A) tailed qRT-PCR of *miR156f*. The qRT-PCR was performed on the PikoReal real-time PCR system (Thermo Fisher Scientific, Waltham, MA, USA). The primers of the housekeeping gene (*protein phosphatase 2A*, *PmPP2A*) [41], *miR156f* [25], and *PmSBP1*/*6* [21] of *P. mume* were referred to in the previous study. The reaction with 10 µL volume (1 µL of cDNA, 5 µL of SYBR Premix ExTaq II (Takara), and 0.2 µL of each primer) were conducted as follow: 30 s in 95 °C, 40 cycles of 5 s in 95 °C and 30 s in 60 °C, and finally end in 20 °C, and each reaction was repeated in triplicate. The qRT-PCR primers of the housekeeping gene (*NtActin*) and eight endogenous flowering-related genes in tobacco used were also from the previous study [42,43,44]. The relative expression level was calculated by the 2^−^^∆∆^^Ct^ method [45]. The error line was drawn according to the standard deviation (calculated by EXCEL) of the three technical repetitions. The significant difference analysis of the gene expression level under GA3 treatment was performed by F-test in EXCEL.

### 4.5. Subcellular Localization

To analyze the subcellular localization of *PmSBP1*/*6*, the CDSs of *PmSBP1*/*6* were cloned into the vector pSuper1300-GFP between *Sal* I and *Spe* I restriction sites to generate the *PmSBP1*/*6-GFP* fusion gene driven by *CaMV35S*. The recombinant plasmid was transformed into *Agrobacterium tumefaciens* GV3101, cultured in 15 mL liquid LB medium (with 50 mg/L kanamycin and 50 mg/L rifampicin) until the OD_600_ = 0.8, then diluted to OD_600_ = 0.5, and injected into the leaves of 4–6 weeks old *Nicotiana benthamiana*. After injection for 24–72 h, the leaves were sectioned and stained in 4′,6-diamino-2-phenylindole (DAPI) solution for 10 min, and observed under TCS SP8 (Leica, Wetzlar, Germany) confocal laser scanning microscope. The fluorescence of GFP and DAPI were detected at 488 nm and 405 nm excitation wavelengths, respectively.

### 4.6. Promoter Activity Analysis

To analyze the promoter activity, the 2000 bp promoters of *PmSBP1*/*6* were inserted into the vector pCAMBIA1305.4-*GUS* through *Sal* I/*Bam* HI restriction sites. After instantaneous transformation in *N. benthamiana* leaves, the leaves were cut and stained in histochemical GUS staining reagent for 24 h, and then rinsed in 75% alcohol to remove chlorophyll.

### 4.7. Overexpression of PmSBP1/6 and Their Promoters in Tobacco

The *miR156*-sensitive gene *PmSBP1*, *miR156*-insensitive genes *PmSBP1tb*, and *PmSBP6* were cloned into the plant expression vector pSuper1300 using *Spe* I/*Kpn I* restriction sites under the *CaMV35S*. *PmSBP1tb* is a synonymous mutation in the *miR156* binding site of *PmSBP1*, and this mutation was completed by Sangon Biotech Company (Shanghai, China). The recombinants *35S::PmSBP1*/*6* and *35S::PmSBP1tb* were transformed into *A. tumefaciens* GV3101, respectively. The *A. tumefaciens* contain *35S::PmSBP1*/*6*, *35S::PmSBP1tb*, and *PmSBP1*/*6-promoter::GUS* vectors were separately cultured in 30 mL liquid LB medium (with 50 mg/L kanamycin and 50 mg/L rifampicin) until the OD_600_ = 0.6–0.8, then diluted to OD_600_ = 0.2–0.5 and infected tobacco by leaf disc method [46]. The phenotypes of T_3_ generation of *PmSBP1*/*6* and *PmSBP1tb* transgenic seedlings were observed and photographed. To determine the tissue expression specificity of the promoter of *PmSBP1*/*6*, different tissues of T_1_ generation of *PmSBP1*/*6*-promoter transgenic seedlings were stained in GUS solution for GUS activity detection.

### 4.8. Phenotypic Statistical Analysis

The phenotype changes of these transgenic and wild-type tobacco were observed, recorded, and photographed. The flower morphology was observed throughout the flowering period, and the anthers were collected when the flower was just opened in each transgenic line. The 2,3,5-triphenyl tetrazolium chloride (TTC) staining and pollen germination in vitro were used to test the pollen viability. Three plants were used in each transgenic line. The TTC staining and pollen germination were observed under the microscope. The number of pollen grains in each field was more than 100, and six fields of each plant were counted. The average value and standard deviation of the three technical repetitions were calculated by EXCEL, and the error line was drawn according to the standard deviation. For transgenic plants of *PmSBP1* and *PmSBP6*, artificial self-pollination was performed at the beginning of flower blooming. The pods were harvested when their color turned brown and cracked. The seeds and pods were photographed, and the seeds were stored in bags with desiccant.

### 4.9. Yeast One-Hybrid Assay

In the cloned promoter sequences of *PmSOC1s*, there are several ‘GTAC’ SBP binding sites. Fragments of about 100 bp including the SBP binding sites during the 850 bp promoters were inserted into the *Sac* I/*Sal* I-cleaved pAbAi vector as baits. The prey vector pGADT7-*PmSBP1*/*6* was constructed by *Bam* H I/*Eco* R I sites. The primers were designed by Oligo7 and shown in Supplementary Table S1. The bait recombinants plasmid was transformed into the yeast Y1HGold strains after being digested by *Bst* BI, and their tolerance to Aureobasidin A (AbA) was detected in the SD/-Ura/AbA medium. Subsequently, the prey vector was transferred into the yeast-containing bait vectors by the Quick & Easy Yeast Transformation Mix (TaKaRa) following its procedure, which was selected on the SD/-Leu/AbA medium.

## 5. Conclusions

Overall, *PmSBP1*/*6* were important regulatory genes in flowering time and fertility, negatively regulated by *miR156* and exogenous gibberellin. Meanwhile, PmSBP1 can directly bind to the promoters of *PmSOC1-2* and *PmSOC1-3* to regulate flowering. In conclusion, a miR156-PmSBP1-PmSOC1s pathway was formed to participate in the regulation of flowering time in *P. mume*. This study lays a foundation for revealing the molecular mechanism of flowering time regulation in *P. mume*.

## Data Availability

Not applicable.

## Supplementary Materials

The following supporting information can be downloaded at: https://www.mdpi.com/article/10.3390/ijms231911976/s1.
