# Supplementary material for: The miR156-Targeted SQUAMOSA PROMOTER BINDING PROTEIN (PmSBP) Transcription Factor Regulates the Flowering Time by Binding to the Promoter of SUPPRESSOR OF OVEREXPRESSION OF CO1 (PmSOC1) in Prunus mume
Source: Int J Mol Sci. 2022 Oct 9;23(19):11976. doi: 10.3390/ijms231911976 (PMC9570364; doi:10.3390/ijms231911976)
Supplement: Supplementary file 1 [file ijms-23-11976-s001.zip › Supplementary Data S1.pdf]

Supplementary Data S1

The cloned promoter sequences of *PmSBP1/6* and *PmSOC1s*

> *PmSBP1-promoter*

ATTGATGAATGCATAGATTGACAGAGAGGGAGAGAGAGAAGGGTAGTTTCTAAAGTAGTGGTAGTGAGA
GAGTGATAAAGGTGGTGAATGTGGAGGGGTAGTGAAAAGCAGACACCTGACAATGGCCCATGCCAGCT
CTGAGCCTCTTAGAGAGAGAAAGAGAGAGGCTTTGTTGGTGCAAAATGTGTGTGTGAGAGTGAGAGAG
TGAGGTACACTGAAAGACTCTGACTGTACAAAAATAATGGAGCAAAACAGCAGAATAGAGAGAGAGA
GACGAATGAATGGATGGGTGCTTTATGGAGCTATCCCTCTCTCTCTCTCTCTCTCTCTGAGCCACTCAGCT
TTACAAGAAGCTCCTTTCACTGTAGCTCTCTCTCACCTGTTTGTTTTTTTTATACACAACTTTCTTCTCTTTT
GAAAGATTTTATAAGCTTTTCCCTTTGCTGGTTATGGTATGGATATGGGTGCCCATACTCATGATGCATGGT
GCCCATCGTTTCCCATTAGCTGGTGCCAAATGTCTAATTTGCCCCTGCTTTCTAGATTATTGTGTTTACATTA
TGATTTACCCCAGATATAAGAAGGATAATACATATGAGTGACTAAAAGATCTCAAATTTGGTTGATTGTTT
TTTCATAGAGGATCTTTAAATGATTATTTAAAAAATAAACACTTGGAATTATAATATGTGAATAGGGAAAA
TTTGATAATTCCAATGTGGGGGACAAGGAGGTACCCAAGTGACAAAGGCATTGTCCTTATAAGAAAGTCT
CTATTGCATCTCGTATCAATTACCGACTAGTCATTTTCAAATTGATTCAGTAATAAACTTCATAAACCGCAT
AATCAAATTCACTAAAATAATCTAAAAATGTAATTTCTCTAGCATTACTCTTAATTGAGTCACAACACAAA
AGAATTATTATTATTTTATTTAGGTAGAAGTACAACAGGATTTGGTAGTTGATTAGTACGAGTTAGTTGGTT
AATTAAAGAAAATAAAAGATGGTAGATGGTAGGATCTATATGCATAGAAGACTGCTGGTCTATATATAGTG
AAGTTAATGCTGTCCGTACATTAATAAGCTGTATCTATGCTTGTAGTGTTAGTCTTGTTTTGGTCAATGATGT
TTGGTTGTTACTAACCTGTTTTGGGTGGCACACACTCGTGTTTCTTAAAAGGGTCACAAATTCATTAGATA
AAGCTTTGATGTACTTGAGTAGGACCTTCACTCATATGCCTGCAACTATTCAATTAGGTAAAAAACAAAA
TCACGTACTGCAATTGGTTTATTTGAACATTTGAATTTCTAATTAGGGAAGGTAGTTACCAAGGTGGATAA
AGGCATCTGAGGT

> *PmSBP6-promoter*

AAGGCAAGAAATAATGAGAGAGAAAGAGAGATGGAAGAGTCAAAGAAGAAGAAGACTAAGACTAAA
ACATTGATATAGGAAAGGGTTTTGGAAAAGAAAGAGATTTATGCGCGAGGACCACAAGTTCCAAATGTT
CTCTTGTCACCACAAGGATAACAGTGAATGGTAGCTAGAAGCCTACAAAAGCCAAAGCTAAGCTCATTT
CCTCCAACAAAACCACATCATCATGTACATGATGTGTACAGCTACAACAACACAACAACAGGCACCAAC
TCTTTTACTGTAACTTTAATTCACATAGACATATTCACATGGTGGCTATTGCACTATCTAATTCGTTTTTAGG
ATAGGTTTTGTGTCTAATCCAATCATGGAGCAAAGGGAATTAACACTTGTCCAATATTGGTTGAACAACC
CAATATTGTTGTACAAATGGGATTTGATTTACAGGGAGATTCAATAAGTATTTAAGTTAAGGATTTTTATA
TATTTCTAATCTTAGTTGTCGGATTGCATTCATTAGATGTGTTGATAGATTTAATATGTAACATACATACATG
CAATCCAACAATCGAGATTAGAAATATGTAACCAATATCCTTATCGGAGAATTTTCCTTGATTTACATATCT
GTCATTTTGCTAGAAACCTGAAACCGAATAAGCCAGTCAGTCAGTGAATCATATAAATATACAGGGTTAG
GTTTAAGTATATAGATTATATATATTTCCGAGGCAAGAAAGAAATGCAGTTGAGGCTTTGTCCTTATAATAG
TTGACAGTCCTTTTCTTGAGAGCGCGACTATTCCTTCATCCACAATGTATTATTGGAAAGTGAAAGGTGAA
AAGAAAACCCTACCTATATGGTCCAATGATGTAAGGAGAGAGCCCTATCATGCTTTGACACATATGGTAC
TATTATCTATTATTTCATGTCCTTGAATCTCATATTTGTTGGCCCTTCTTTCCTTTCCTTTGTTTTTACACTAAC
ATTATGGTGTTAGCCAACTGGTTTGCAGCAAACAAAGTGGCCAATTTCTGTTACCAATTTATGTCAATTGT
TGCTAGCAAGCAGCTTGGACCACACTTTCATACGTTGCAATTTATAAATATTGGTCCCAATATGTACGACC
CAAGACAAGAGCTACCGCTAGCATGTTTTTTTCTGCAACATGGACAGGATGCATGGTTTAATTACCAATTA
ATCAACTATTATCCAAGTTTTAATTACTAATTAGTATGAAAGTTCAATGTATATATAAACAGAGTTTGGAAT
TTGAAAACTCTAAAAGTGGGATGTGAAATAAAAGTCAGTGAAGTTATCGAGTCGGGAAAAAGTTGTTGC
CGTAAGGTATAATTGAGTAGAATCTAGTGGAGGAGCCTTTTGAAGAAAGGACAGCATGCATCATGGTTTC
ACGCATCTCATAAGGAGGCCACAACTTTCTATCTAGGGAAATTTTTTTATATGTTTCGAGATGGGACATGC
GGAAATATTCTATACCAAAATTACTCATGTTTAAGACGGTAAATTGGTTCTATACAATAGTTGGAGTTCGA
GTACGACTCGCATTTGGCTCGTTTGGTTCACAAATAAGCCGAGCTGAGTTCGAGCTTGAAAAAATCATTT
TGACCCAAACTTAAGAACTTTGGTATTTGATCTCCCAGAATTTGATTTTGGTTTTGGTCTCATTCATGTATAT

ACTAGCACTCCTGCACTAGAAGAAACTTGAAGCCCTAATATTGACCAGCTAATCCACTAGATA

> *PmSOC1-1-promoter*

TAACAGAAGGAAGCGCTCTCAGCTTTCACTGCACAAATCTATTCAGAACCCAATGTGTCAAAAAGTGAT
GAGAGTTGAGAAGAAGGTGAAGAAGTTAATTGAATCTTACCCAATAGGTAAAATTAACCCAAGAAGCTT
TTTTTGGGTTTGTTTTGTGGAAGAGAGAGGTTTGTGACTTGTGGGTAGGACTTGGAAAGATGATAAAGAT
GAGGTTAAATATATTGGATCCTCTTCTTCAATTCAAATAAGCAATCAATATATAGAATTTGTGTAAAGGAA
ATATGAGTTCTTTAAGTTCTTTAATCAAAATGAAGATATGAAGGAGAGAGAGAGAGAGAGAGAGAGAGA
GAGAGAGAGAGGCAAAGGGGAAGAATTGAGTGTTAAGAAATTGGCCCCAAATAGCCTCTTGGTTACAA
ACAAAAGAAGGAAGAAGAAGCCCAAAATGGAAAGAAAAGAAACTAAAAGAGACCTTCTCAGGTTTGT
GAGCAAAGACTTGGACTAGACCCTGAAAAATACAAATATATATATATATATATATTACATATAATAGGTGG
GTTTTTGTGATTGATTGATTCAACTCCGAGAACCAAAAGACAAAGCAAAACAGAAAACTTCTTCAGCTC
AAATCTCCTCAACAAGCTAAAAAAGGAAAAACAGAGTTCAAGAGAAGCTGAGCCAATTTTCCAAATAT
GCAAAAGGAAACCCAGAAAGAAGCTATGGACAAATAAGGGAGGAAGAAGATGCCAAAGAAGGAAAG
AGAGAGAGAGAGAGAGATGAGGAGGATTGAGAGAGGGAAACCACAAAGACCCAGAAATTATAGAGA
GAGTAGGAAGGAAGATTTTGA

> *PmSOC1-2-promoter*

AGAGATGAATAAAAACGAAAGCCATTTTTTTATTCCCAATGCGGTTTCCGAAAAAGATGAATAAAAAAA
AATATTCCCTCTTTTACTTCTTTGTTTCTTAATTTTGCCAAAAAAGAGAAGCCATGAGCATTTAAGTAGTGT
TTTATCTAATATTTTCACTGCGTTTTCTTTCCTCTAATTCTTATCCCTTTCTTTTTTAGTGGGGTAGAATTTTAA
AGACAAAAGAAGGCATAGAGAATAATGGCAGTGAGTTATCGATCAATATGGAAAAAAGGGGTCTGGTG
GCCTGAGGATAAAGAAATCGGAACATCCACAATCAGATAAAAAGAAAGTTCTTCCCTTCTGCACTGCAC
TGTGGACTATTTCTAGGGTTTCTGATCTATTTTCCATATTTGTATTCTATCATACTCCTATACCCTCGTGTTGT
GTCCTTTGTTGCTCTTTTTGGGTAATTCATGTCTATGGGTGGCGTCTCTGGGTTCTATTTTTAGAAATCTAAT
TTAACATATCTTTTTTGGGGGCTTTCTTTTATGAATCCTCCTGGGGTTTCATCTGCTAATTGCTTCTTCAGAG
CCAAAGTCTGATACTTTGTTTGGATTTTCTAAACTAAACCTCTCCTGGGTTTGCTTGGTTGTCAAAGGTCCT
TCACAAATTTCTACATTTTTCTCTACTAGCTAGTGTTCGAGCACTTCAAATCTTCGAGGGGGTTTCCAGGT
GTAACTCTTCTCTACTAGCTTATTTCTTGAGCTATATACATGTATAGCGCATCTTTACCAATTTTCATGGTATT
ATTACTTTTATTATTATCATCTCACGATGATCTTGTAATTAATGATGGCGATGATCTAGGCT

> *PmSOC1-3-promoter*

CCGCTTCCAAACAAAGAAAAGCCCCAGAAAATCAAATGGTTTCCAAAACCCAGAAAAGAAAAGAAAA
AAGGATGAAGAAAGGTTTCCAAAAAATAGAAAGGGGGCAGACCAAAATCCAAGATAGATATATGTGAAA
CGGAGAAATCCCAGAAACCCTAGGGGAAAATTTGAGGAGAGAGATGAAGAAGGGGGAGGAGGAAGAT
CAACAAAAAATTGATGGATTTGCCCTACTTGCTTCTTTCCCTTTTTAGGTTTCAAAGTCTACAAGAGAGAA
CCCTATGAGGTATATTTGAGGTAATATAAAAAGATGGAATAAAATGAAAAAGGAAAGGAGGACATCAAC
CCAATCTACAACCCCTTTCTTTTTTTGGCAATTAATGGACACCTCCCCATAAAGAAAAAAAACAAGAGAA
TAAATGCCTAAGGCTGAGCTTAGCTTACACATGCGAAGCCACTTACTATTCCTCCACCCAATGGTAAAAA
TAAATATATTAAAAACCTTTATTGGAGGCACACAAACTCTGTGCTATCAAGTCTTTTGGTGACACGTGTGT
AAAGTGACTGACAACCCTCTGTGGCCGGTGACTGTTTGAGCGGGCAGAGAGGCGCTTGAAAGCTAAAA
CAGCGAACATTCCTGTCGGTTGCCAGTTAAATTCAGACAGTCGATATAACCCCAAAGTTGCAGGTAAAAT
TTGGGGCATCGGATGGGCACAGTAAAAAAATTCTGTAAATAATGACTTGTCCGTACAATGGTTGAGATCT
CCTAGCAAAAAAAAATGACAAGGCAATTTTCCGTACGATTGATAGAAGAGATGGGCTAATGGGGTGATG
CCACATGCCGCCAGAACTCAACACATCACGG
