# Supplementary material for: The miR156-Targeted SQUAMOSA PROMOTER BINDING PROTEIN (PmSBP) Transcription Factor Regulates the Flowering Time by Binding to the Promoter of SUPPRESSOR OF OVEREXPRESSION OF CO1 (PmSOC1) in Prunus mume
Source: Int J Mol Sci. 2022 Oct 9;23(19):11976. doi: 10.3390/ijms231911976 (PMC9570364; doi:10.3390/ijms231911976)
Supplement: Supplementary file 1 [file ijms-23-11976-s001.zip › Supplementary Figures.pdf]

Supplementary Figures

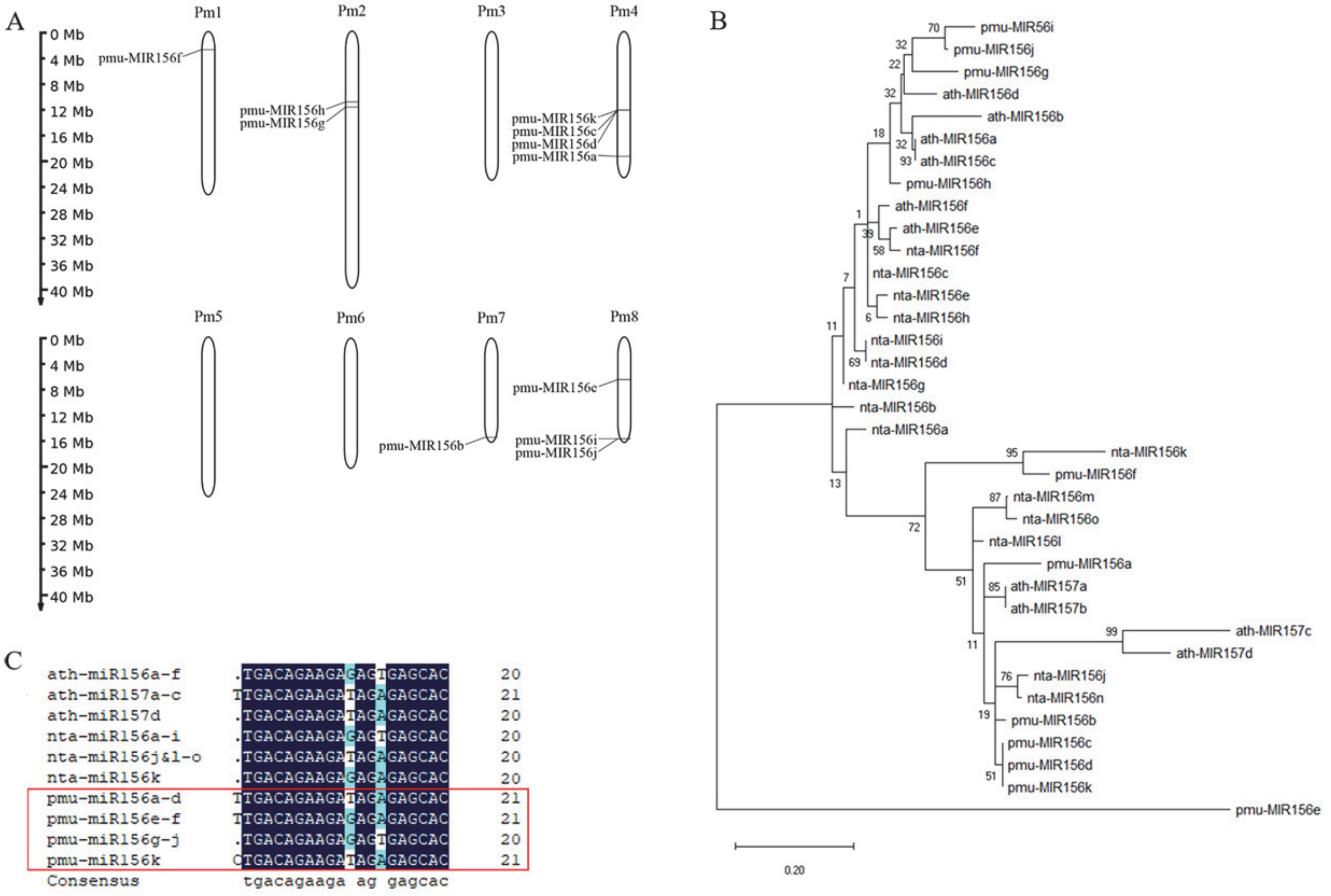

Supplementary Figure S1. Sequences analysis and phylogenetic analysis of miR156 family in *P. mume*. (A) The chromosomal location of the miR156 family in *P. mume*. (B) The phylogenetic tree of miR156 precursor sequences from *P. mume*, *Arabidopsis*, and tobacco. (C) Alignment of the mature miR156 sequences from *P. mume*, *Arabidopsis*, and tobacco.

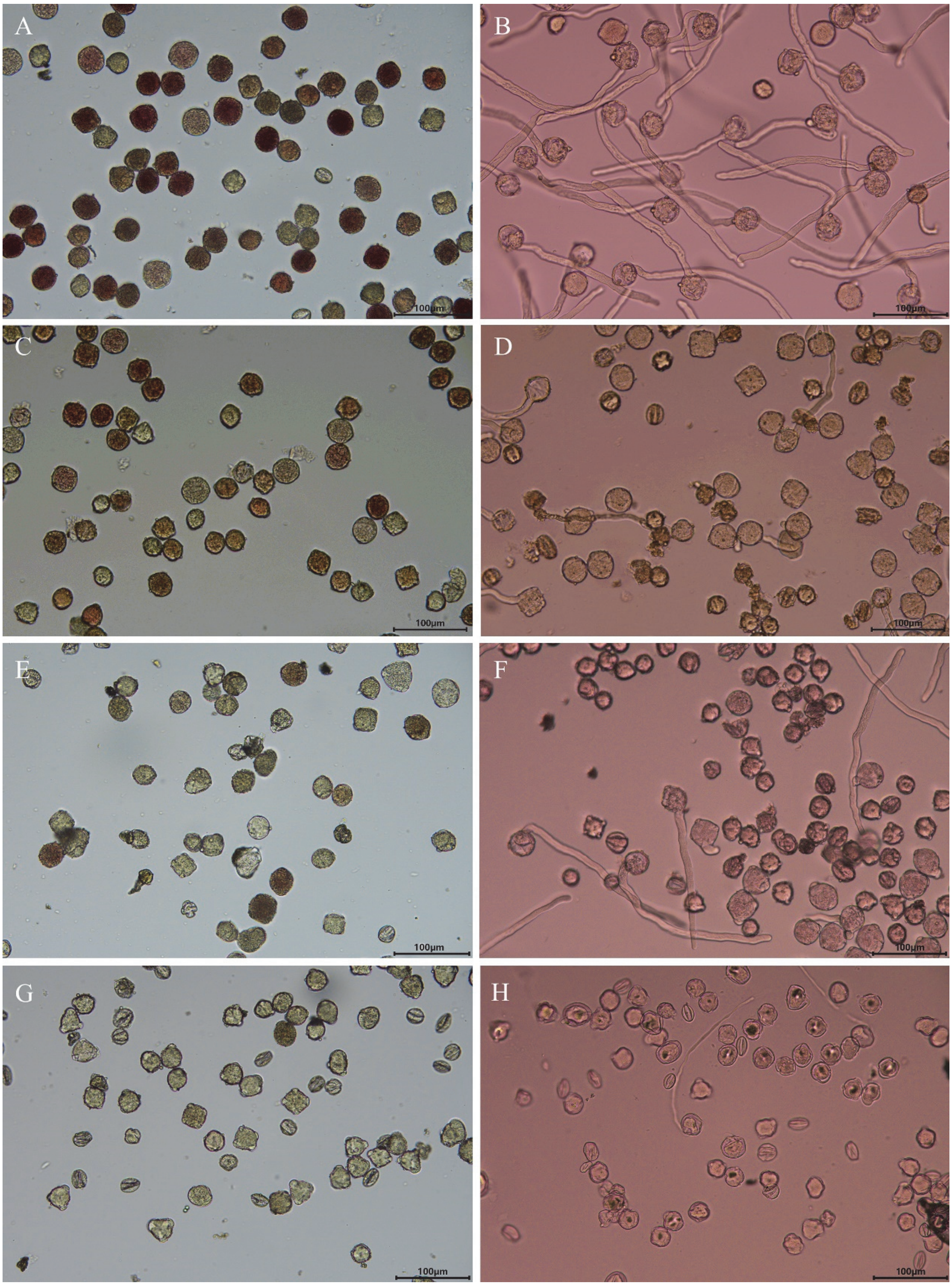

Supplementary Figure S2. The Pollen viability and pollen germination of *PmSBP1* transgenic tobacco. (A) The pollen viability of wild-type tobacco. (B)The pollen germination of wild-type tobacco. (C&E&G) The pollen viability of *PmSBP1* transgenic lines S1-3, S1-5 and S1-6. (D&F&H) The pollen germination of *PmSBP1* transgenic lines S1-3, S1-5 and S1-6. Scale bar=100 μm.
