# Supplementary material for: The miR156-Targeted SQUAMOSA PROMOTER BINDING PROTEIN (PmSBP) Transcription Factor Regulates the Flowering Time by Binding to the Promoter of SUPPRESSOR OF OVEREXPRESSION OF CO1 (PmSOC1) in Prunus mume
Source: Int J Mol Sci. 2022 Oct 9;23(19):11976. doi: 10.3390/ijms231911976 (PMC9570364; doi:10.3390/ijms231911976)
Supplement: Supplementary file 1 [file ijms-23-11976-s001.zip › Supplementary Tables.pdf]

Supplementary Tables

Supplementary Table S1. The primers designed by Oligo7.

Primer name	Sequences 5′-3′	Used for
PmSBP1-F	ATGACAATTATGGAAATGGGC	gene clone
PmSBP1-R	TTAAAGTGACCAGTGAACATG	
PmSBP6-F	ATGGAATCAAACAGAGCTCATGGG	
PmSBP6-R	TCATTCTCCATGATATTCAGAGGAGGTTT	
PmSBP1-2M-F	ATTTACTGAATTGTGCTCTC	promoter clone
PmSBP1-2M-R	TGTCATTTACAGCCCATT	
PmSBP6-2M-F	CACCGAATGTGTTTTTGCCTCA	
PmSBP6-2M-R	TTTGGAGAAAATTAGGAGAGAAGGCAAG	
PmSOC1-1-1M-F	TCAAAATCTTCCTTCCTACTCTC	
PmSOC1-1-1M-R	TAACAGAAGGAAGCGCTC	
PmSOC1-2-1M-F	AGAGATGAATAAAAACGAAAGCCA	
PmSOC1-2-1M-R	AGCCTAGATCATCGCCAT	
PmSOC1-3-1M-F	CCGTGATGTGTTGAGTTCT	
PmSOC1-3-1M-R	CCGCTTCCAAACAAAGAA	
gus-*SBP1-2M*-F	TTATCGATACCGTCGACATTTACTGAATTGTGCTCTC	vector construction of *35S:PmSBP1/6-promoter-GUS*
gus-*SBP1-2M*-R	TGACCACCCGGGGATCCTGTCATTTACAGCCCATT	
gus-*SBP6-2M*-F	GCTTATCGATACCGTCGACCACCGAATGTGTTTTTGCCTCA	
gus-*SBP6-2M*-R	ACTGACCACCCGGGGATCCTTTGGAGAAAATTAGGAGAGAAGGCAAG	
my-*SBP1*-F	GTCGACATTTAAATACTAGTATGACAATTATGGAAATGG	*35S: SBP1/1tb*
my-*SBP1*-R	AGCTTTTGCTCCATGGTACCAAGTGACCAGTGAACATGCT	
pc-*SBP6*-F	ACGGGGGACTCTTGACCATGGATGGAATCAAACAGAGCTCATGGG	*35S:SBP6*
pc-*SBP6*-R	GGGAAATTCGAGCTGGTCACCTCATTCTCCATGATATTCAGAGGAGGTTT	
gus-PmSBP1-F	CGGGGGACTCTAGAGGATCCATGACAATTATGGAAATGGGC	*35S:PmSBP1-GUS*
gus-PmSBP1-R	GACTGACCACCCGGGGATCCTTAAAGTGACCAGTGAACATG	
pAi-*OC2-B1*-F	AAGCTTGAATTCGAGCTCAGCTTATTTCTTGAGCTAT	pAbAi-Bait
pAi-*OC2-B1*-R	ACATGCCTCGAGGTCGACTTAATTACAAGATCATCGTGAG	
pAi-*OC2-B2*-F	AAGCTTGAATTCGAGCTCTTCTATCATACTCCTATACCCT	
pAi-*OC2-B2*-R	ACATGCCTCGAGGTCGACAGATTTCTAAAAATAGAACCC	

pAi-*OC2-B3*-F	AAGCTTGAATTCGAGCTCATTCCCTCTTTTACTTCT	
pAi-*OC2-B3*-R	ACATGCCTCGAGGTCGACGAAAGAAAACGCAGTGAA	
pAi-*OC3-B1*-F	AAGCTTGAATTCGAGCTCGCCTCCAATAAAGGTTTT	
pAi-*OC3-B1*-R	ACATGCCTCGAGGTCGACATAAATGCCTAAGGCTGA	
pAi-*OC3-B2*-F	AAGCTTGAATTCGAGCTCGCATGTGGCATCACCCCA	
pAi-*OC3-B2*-R	ACATGCCTCGAGGTCGACTGTCCGTACAATGGTTGAGA	
AD-*SBP1*-F	ATGGAGGCCAGTGAATTCATGACAATTATGGAAATGG	pGADT7-PmSBP1
AD-*SBP1*-R	CTCGAGCTCGATGGATCCTTAAAGTGACCAGTGAACATG	
AD-*SBP6*-F	ATGGAGGCCAGTGAATTCATGGAATCAAACAGAGCTCATGG G	pGADT7-PmSBP6
AD-*SBP6*-R	CTCGAGCTCGATGGATCCTCATTCTCCATGATATTCAGAGGA GGTTT	

Supplementary Table S2. Details of the *cis*-acting elements of the promoter sequences of *PmSBP1/6*.

**Gene name**	***cis*-acting elements**	**Sequences 5′-3′**	**Amount**	**Function**
PmSBP1	TCT-motif	TCTTAC	1	light response
	LAMP-element	CTTTATCA	1	light response
	CAG-motif	GAAAGGCAGAC	1	light response
	Box 4	ATTAAT	2	light response
	G-box	CACGAC	2	light response
	TCCC-motif	TCTCCCT	1	light response
	GT1-motif	GGTTAAT	1	light response
	TCA-element	CCATCTTTTT	1	salicylic acid response
	ABRE	ACGTG	1	abscisic acid response
	ARE	AAACCA	2	anaerobic induction
	GARE-motif	TCTGTTG	1	gibberellin response
	GCN4_motif	TGAGTCA	2	endosperm expression
PmSBP6	LAMP-element	CTTTATCA	1	light response
	Box 4	ATTAAT	1	light response
	TCCC-motif	TCTCCCT	1	light response
	AE-box	AGAAACTT	1	light response
	chs-Unit 1 m1	ACCTAACCCGG	1	light response
	ACE	GACACGTATG	1	light response
	GATA-motif	GATAGGG	1	light response
	ATCT-motif	AATCTAATCC	1	light response
	MRE	AACCTAA	1	light response
	GA-motif	ATAGATAA	1	light response
	ARE	AAACCA	5	anaerobic induction

MBS	CAACTG	2	drought-inducibility
P-box	CCTTTTG	1	gibberellin-responsive
O2-site	GATGATGTGG	1	zein metabolism regulation
CAT-box	GCCACT	1	meristem expression
