# Supplementary figures and images for: The miR156-Targeted SQUAMOSA PROMOTER BINDING PROTEIN (PmSBP) Transcription Factor Regulates the Flowering Time by Binding to the Promoter of SUPPRESSOR OF OVEREXPRESSION OF CO1 (PmSOC1) in Prunus mume
Source: Int J Mol Sci. 2022 Oct 9;23(19):11976. doi: 10.3390/ijms231911976 (PMC9570364; doi:10.3390/ijms231911976)

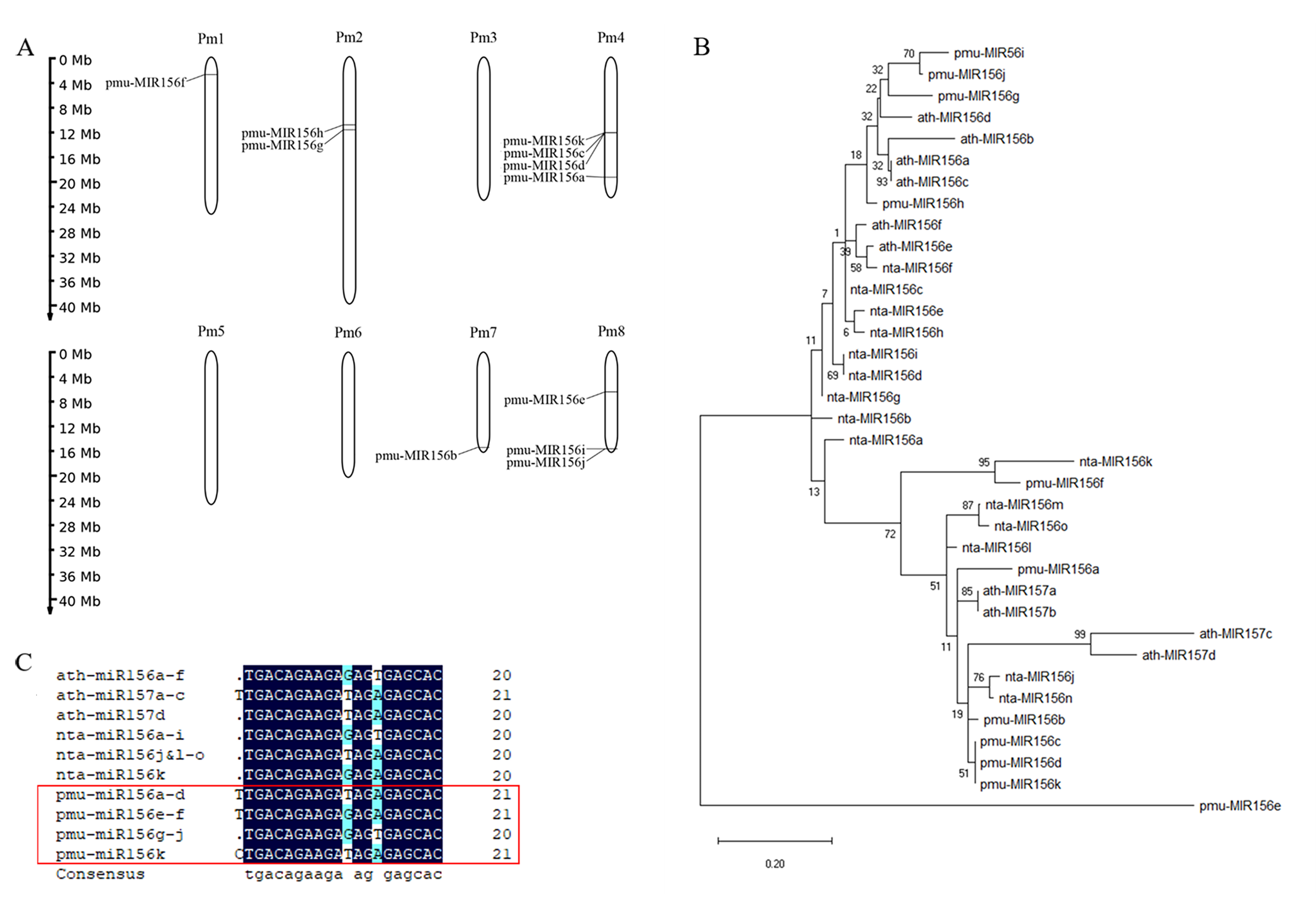

Supplement: Supplementary file 1 [file ijms-23-11976-s001.zip › Supplementary FigureS1.tif]

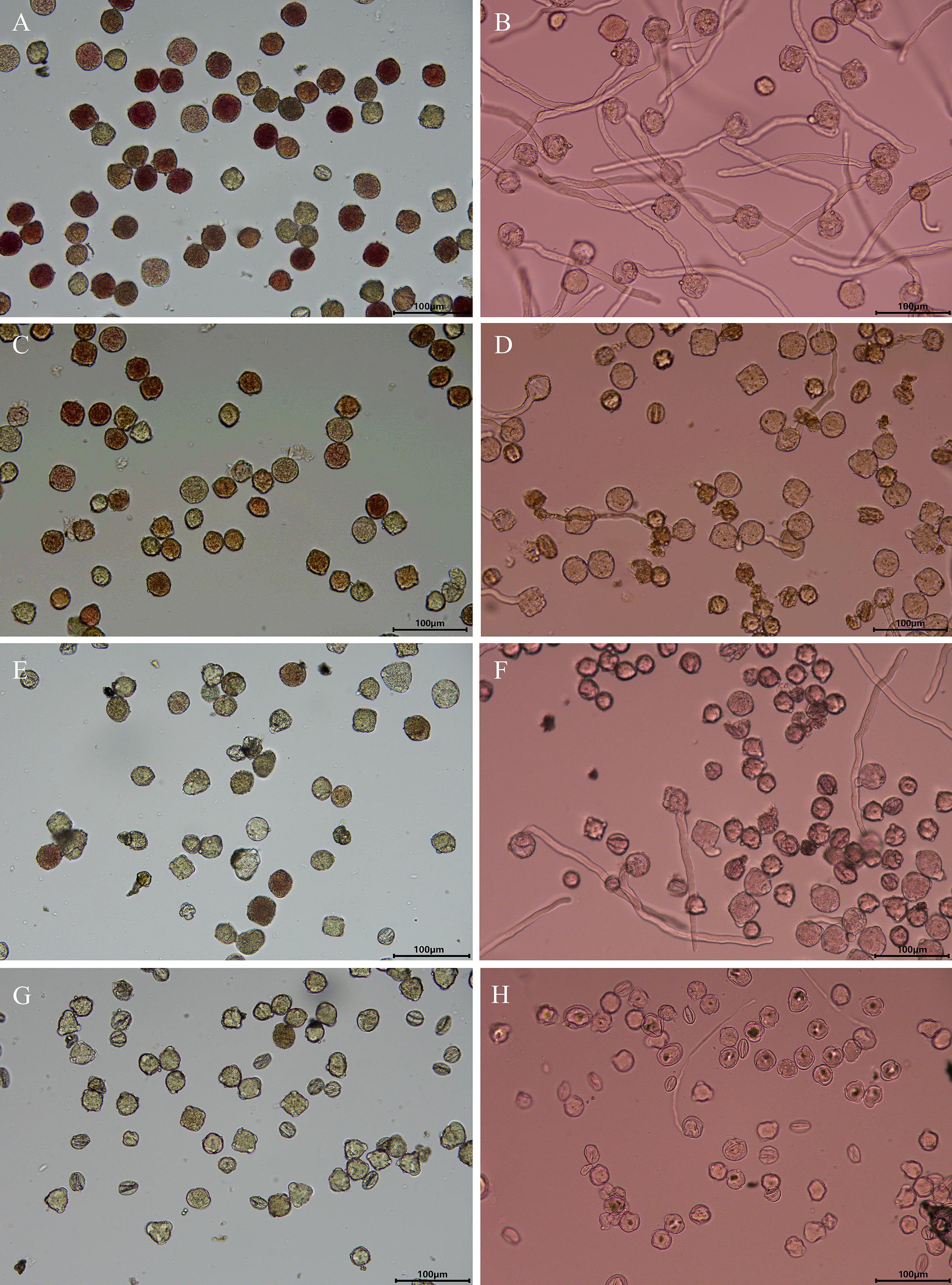

Supplement: Supplementary file 1 [file ijms-23-11976-s001.zip › Supplementary FigureS2.tif]
